# *R*. *nukuhivensis* acts by reinforcing skin barrier function, boosting skin immunity and by inhibiting IL-22 induced keratinocyte hyperproliferation

**DOI:** 10.1038/s41598-019-39831-w

**Published:** 2019-03-11

**Authors:** Florence Abdallah, Gaël Lecellier, Phila Raharivelomanana, Chantal Pichon

**Affiliations:** 1 Centre de Biophysique Moléculaire, CNRS-UPR4301, 45071 Orléans, France; 2 Université de Paris-Saclay UVSQ, 55 Avenue de Paris, 78000 Versailles, France; 3 Université de la Polynésie Française, UMR 241 EIO, 6570 - 98702 Faa’a Tahiti, Polynésie Française; 4 Université d’Orléans, Collegium Sciences et Techniques, 45100 Orléans, France

## Abstract

*Rauvolfia nukuhivensis* is a well-known plant used for its wide range of beneficial effects in Marquesas islands. It is made up of diverse indole alkaloids and is used as traditional medicine for skin application. The actual mechanism behind the virtue of this plant is still unknown. Hence, in this study we aimed at deciphering the impact of *R*. *nukuhivensis* on skin immune system in context of (1) homeostasis, (2) pathogen infection and (3) inflammation. Here we show that *R*. *nukuhivensis* enhances cellular metabolic activity and wound healing without inducing cellular stress or disturbing cellular homeostasis. It reinforces the epithelial barrier by up-regulating hBD-1. Nevertheless, in pathogenic stress, *R*. *nukuhivensis* acts by preparing the immune system to be reactive and effective directly. Indeed, it enhances the innate immune response by increasing pathogens sensors such as TLR5. Finally, *R*. *nukuhivensis* blocks IL-22 induced hyperproliferation *via* PTEN and Filaggrin up-regulation as well as BCL-2 downregulation. In conclusion, this study provides evidence on the several cutaneous application potentials of *R*. *nukuhivensis* such as boosting the immune response or in restoring the integrity of the epithelial barrier.

## Introduction

Natural products play an important role in the field of new drugs research and development. Traditional medicine has a long history of serving civilization all over the world. Ancient ancestry believed that a disease has a natural cause, so natural herbal remedies can treat it. It is only from 19^th^ century that researchers started to isolate the active principle from medicinal plants that became important clinical agents such as ajmalicine and reserpine used for hypertension treatment^1–5^. *Rauvolfia* is a genus of the *Apocynaceae* family characterized by their indole composition^6–9^. *Rauvolfia* genus includes more than 100 species that are native to tropical and subtropical regions of the world. *R*. *nukuhivensis* is one of these endemic species that belong to the Marquesas archipelago from French Polynesia. In traditional medicine, the extracts of this flowering plant are still excessively used for skin issues namely for women intimate care and wound healing^5,10^. The absence of scientific explanation of the plant virtues resulted in its uncontrolled usage that critically endangered this species. In fact, the biological characterization of *R*. *nukuhivensis* extract (RNE) is essential to limit its extinction and to comprehend its impact on the cutaneous physiological-immunological mechanisms. Therefore, this study aimed at defining the role of RNE in skin biology by studying its impact on the skin immune system from cellular activities to innate immune responses in homeostasis, in pathogenic stress and in inflammation.

The study was performed on keratinocytes (KCs) which constitute over 95% of the epidermal cell type and are the first sensors of pathogen invasion^11,12^. Moreover, the skin contains a highly dynamic immune system that is dependent on a cross-talk between KCs and other sentinels of the immune system^11^. This connected network is governed by KCs that are in constant contact with the external environments ensuring skin barrier function and defense mechanisms for homeostasis maintenance. Hence, different cutaneous components are mobilized to preserve the skin integrity through innate immunity, which is the most active and effective actor against daily life external insults challenges^13^. To face that, the skin renews itself each month ensuring by that skin restoration, rejuvenation and reepithelization. This process involves a complex interplay between several cutaneous innate and adaptive actors such as antimicrobial peptides (AMPs), inflammatory mediators and proliferative cytokines such as IL-22. For instance, AMPs generation by the innate immune system is essential for epithelial barrier preservation^14^. In human, β-defensins (hBD) have been identified in skin and constitute the most expressed AMPs^15^. Four types of cutaneous β-defensins (hBD-1, 2, 3 and 4) are differentially expressed either in constitutive or inducible manner^16^. hBD-1 constitutively expressed in keratinocytes is responsible of cutaneous immunosurveillance. However, hBD-2 and hBD-3 are induced upon pathogens invasion and inflammation^17–19^. When the skin is challenged with pathogens, an inflammatory response is mediated by the innate immune system to clear and neutralize them. This pathogen invasion danger is sensed by KCs via their innate receptors such as TLRs enabling innate inflammatory pathways activation. TLR5 is one of the most expressed receptor on KCs surface^20,21^. Indeed, it is activated by Flagellin which is the major constituent of bacterial flagella. The subsequent activation results in NF-κB signaling leading to increased proinflammatory mediators such as CXCL-8, IL-6 and IL-22^22–25^. In some cases, this response becomes uncontrolled and causes cellular processes perturbations resulting in inflamed epidermis. In fact, several skin disorders are characterized by unbalanced KCs proliferation and differentiation caused by increased inflammatory environment enriched by IL-22. This key cytokine acts as a shuttle between leukocytes and epithelia in non-hematopoietic cells at body barriers including epithelial cells of the lung, gastrointestinal tract and KCs in the skin^26–28^. Nevertheless, continuous exacerbated or uncontrolled IL-22 signaling leads to undesirable tissue inflammation, accelerating certain immune pathologies such as psoriasis, atopic dermatitis and rheumatoid arthritis^29–32^. IL-22 belongs to IL-10 family and is mainly produced by CD4^+^ effector T (T_eff_) cells as well as innate lymphoid cells 3 (ILC3)^33^. It signals through the IL-22R1/IL-10R2 complex and subsequent JAK–signal transducer, activator of transcription (STAT) signaling pathways (anti-apoptosis, cell cycle, differentiation…) and the mitogen-activating peptide kinase pathway^34–36^. So, IL-22 has an important role in the maintenance of epithelial integrity at barrier surfaces^37,38^. Its main physiological job includes reinforcement of epithelium barrier function through induction of hBD-2 and hBD-3 in context of infection^14,17–19,26,27,39,40^, wound healing through promotion of epithelial cell proliferation and survival following tissue damage in pathophysiological conditions^41–43^. Hence, the fine-tuning of IL-22 signaling implicated in these processes can be beneficial to treat skin disorders.

The aim of the study is to define the impact of RNE on skin innate immunity in homeostatic and non-homeostatic conditions. To this purpose, we first evaluated the cellular stress induced by RNE through cytotoxicity and oxidative tests. Then, we looked at the wound healing properties by following wound closure over time and skin barrier functions by defining the relative expression of genes belonging to β-defensins family. We assessed the impact of the extract on skin inflammatory response using a variety of assays. Last but not least, we investigated the role of RNE in IL-22 induced proliferation by evaluating gene expression implicated in proliferation processes which are deregulated by IL-22 stimulation.

## Results

### RNE enhances wound-healing properties without disturbing cellular processes

**Figure 1:**
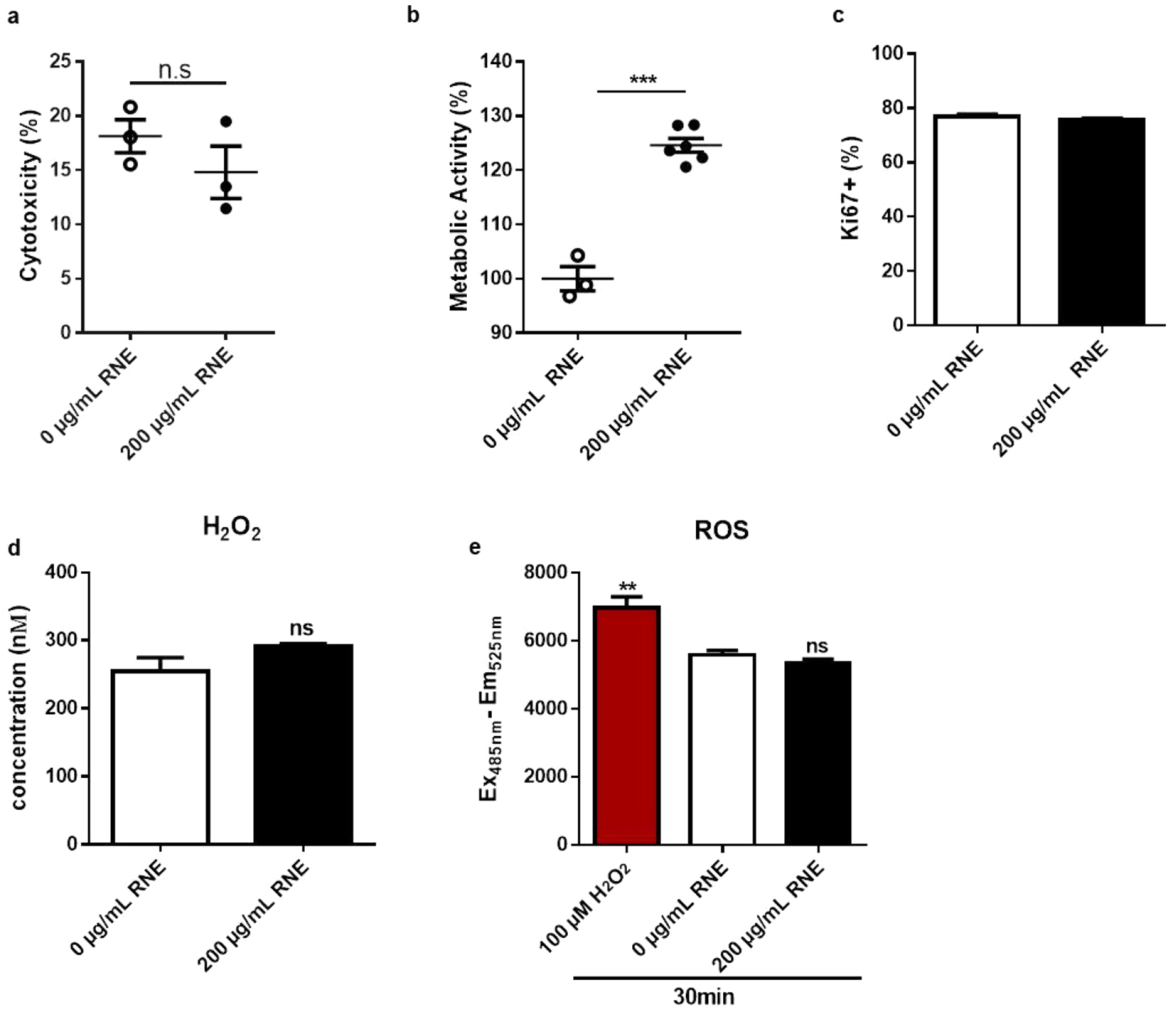
Characterization of RNE in steady state physiology. HaCaT cells were stimulated with 200 μg/mL of RNE (●) or with medium (○) for 24 h, and then cellular processes were evaluated: LDH cytotoxicity assay (**a**), XTT assay (**b**), Ki67 (**c**), H_2_O_2_ assay (**d**) and ROS assay (**e**). *P < 0.05, **P < 0.01, ***P < 0.001, two-tailed Student’s *t*-test. Data are representative of two or three independent experiments with 3 or 5 samples per group in each (mean and SEM.).

**Figure 2:**
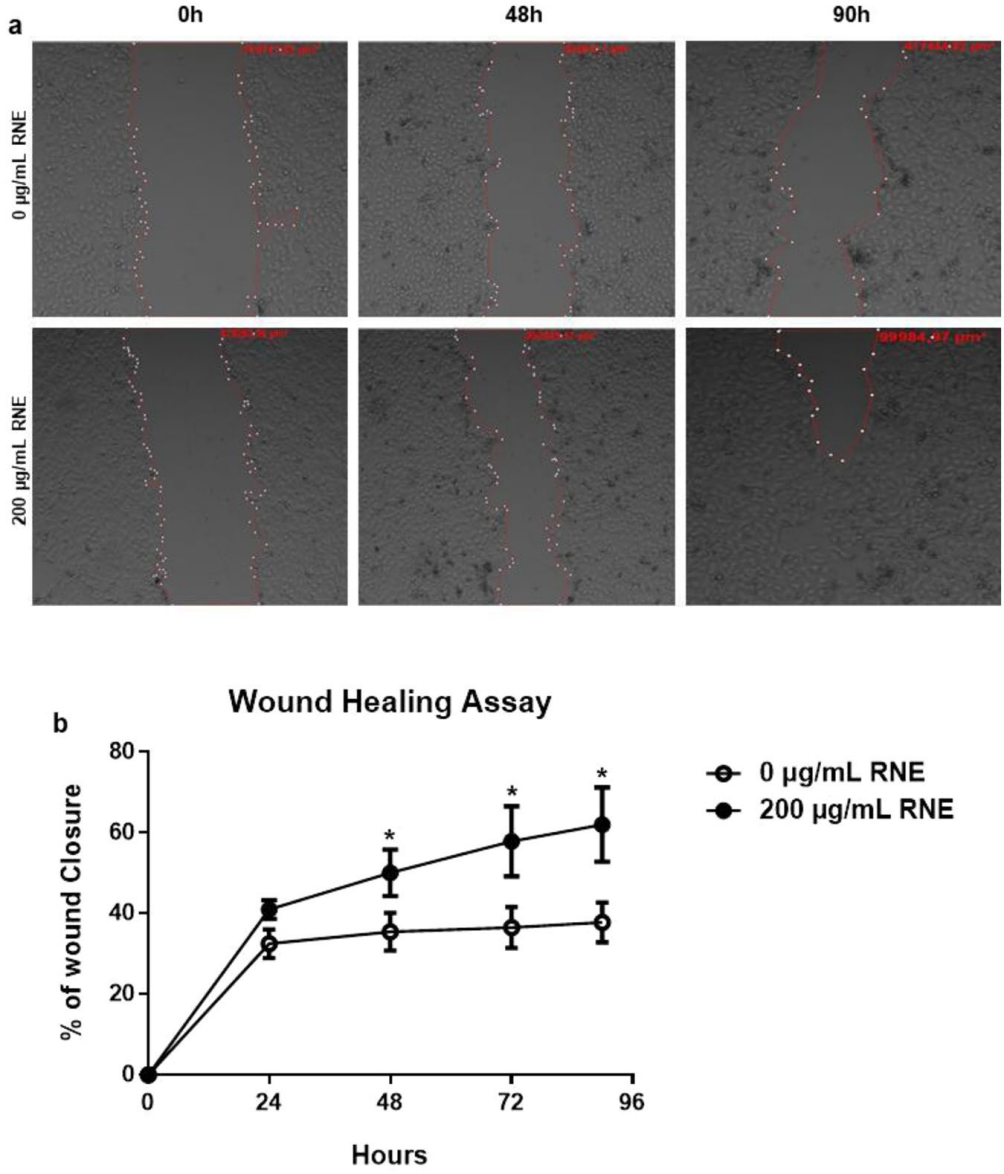
RNE treatment accelerates wound healing. *In vitro* scratch assays assessing the migration rate of HaCaT cells with 200 μg/mL of RNE or with medium during 96 h (**a**). The wound closure area was calculated using Zen 2.3 SP1, Zeiss (**b**). *P < 0.05, **P < 0.01, ***P < 0.001, two-tailed Student’s *t*-test. Data are from two independent experiments done in triplicate (mean and SEM.).

The first challenge was to define the non-cytotoxic effective concentration of RNE to be used since the extract is composed of alkaloids that can be toxic for the cells. Thus, we achieved a kinetic of a dose-response experiment that showed the absence of cytotoxicity and enhanced viability profile below 300 µg/mL for cells treated during 24 h and 48 h (see supplementary Fig. S1). For this study, the concentration of 200 µg/mL was chosen and used throughout the experiments. Then, we investigated the impact of RNE on cellular processes in steady state condition. We checked the metabolic activity by an XTT assay, cellular cytotoxicity by an LDH assay, cellular proliferation by a Ki67 staining, and oxidative stress by ROS and H_2_O_2_ production. The stimulation of HaCaT with RNE for 24 h didn’t have any cytotoxicity effects (Fig. 1). However, a significant increase (around 40%) of the metabolic activity was recorded compared to unstimulated cells (Fig. 1b). Yet, the percentage of Ki67 positive cells was similar in presence or absence of RNE (Fig. 1c). Hence, to verify if RNE causes any cellular stress, we performed a seahorse test that consists of quantifying different parameters of mitochondrial respiration. The seahorse test showed a similar profile between untreated and RNE treated HaCaT confirming by that the absence of mitochondrial dysfunction (See supplementary Fig. S2). Similarly, RNE failed to induce ROS and H_2_O_2_ production showing the absence of an oxidative stress (Fig. 1d,e). Since Polynesian people use RNE to treat skin wounds, we performed a wound-healing assay. One essential step of the healing process is the epithelialization ensured by cell migration, which is the major read out of this assay. HaCaT stimulated with RNE showed a significant increase of keratinocyte migration into the cell free area resulting in almost complete recovery at 92 h compared to unstimulated cells (Fig. 2a). RNE application resulted in 30% wound closure improvement with respect to untreated cells (Fig. 2b).

### RNE impacts on defensin gene expression and inflammatory cytokine secretion

**Figure 3:**
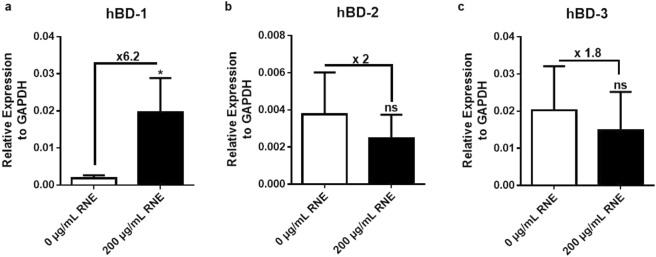
The effect of RNE on cutaneous barrier function. Antimicrobial peptides belonging to the β-defensins family were measured by qRT-PCR after 24 h HaCaT stimulation with or without 200 μg/mL of RNE. hBD-1 (**a**), hBD-2 (**b**), hBD-3 (**c**). *P < 0.05, **P < 0.01, ***P < 0.001, Mann-Whitney test. Data are representative of three independent experiments done in triplicates (mean and SEM.).

Here, we investigated the effect of RNE on β-defensins mRNA expression (Fig. 3). HaCaT were incubated with RNE for 24 h and gene expression was analyzed using real-time qPCR. The results revealed a significant increase of 6.2-fold for the constitutive hBD-1 (Fig. 3a) whereas a decreased tendency profile (~2-fold) for the inducible hBD-2 and hBD-3 (Fig. 3b,c) was observed.

**Figure 4:**
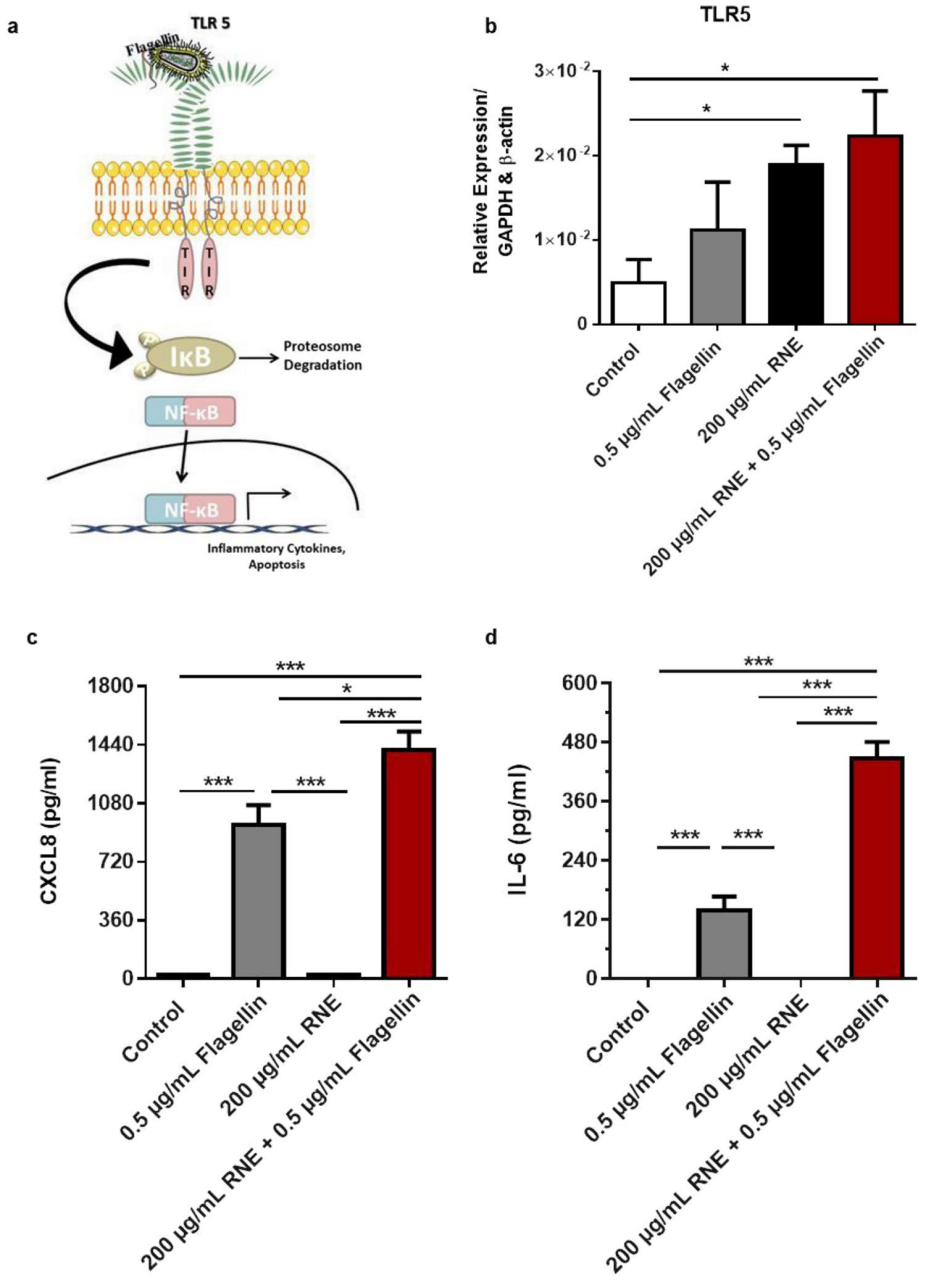
RNE a natural occurring immune adjuvant. Simplified schematic representation of TLR5 pathway (**a**). The relative expression of and TLR-5 was assessed by qRT-PCR (**b**), the inflammatory response was evaluated by CXCL-8 (**c**), IL-6 (**d**) ELISA assays on supernatants from HaCaT stimulated with or without 0.5 μg/mL Flagellin and/or 200 μg/mL of RNE for 24 h. *P < 0.05, **P < 0.01, ***P < 0.001, Two-way ANOVA analysis. Data are representative of three independent experiments with n = 6 replicates (mean and SEM.).

The traditional usage of *R*. *nukuhivensis* to treat cutaneous infections prompted us to study its impact on inflammatory response in presence of Flagellin (TLR5 agonist) a well-characterized inflammatory pathway in KCs. To this aim, KCs were stimulated with 0.5 μg/mL Flagellin in presence or absence of RNE during 24 h. Then, TLR5 mRNA expression was measured by RT-qPCR and CXCL-8 and IL-6 secretion were assessed by ELISA. TLR5 gene relative expression was increased by 2-fold for 0.5 μg/mL Flagellin treated KCs whereas 3-fold increase was observed for RNE treated KCs compared to control (Fig. 4b). Nevertheless, the co-stimulation of KCs with Flagellin and RNE resulted in the highest up-regulation that reached a 4-fold increase compared to basal expression (Fig. 4a). Accordingly, the evaluation of inflammatory mediators showed a significant increased production of CXCL-8 and IL-6 upon Flagellin stimulation while RNE stimulation didn’t induce cytokine production (Fig. 4c,d). The co-stimulation Flagellin with RNE resulted in the highest CXCL-8 and IL-6 production (Fig. 4c,d).

**Figure 5:**
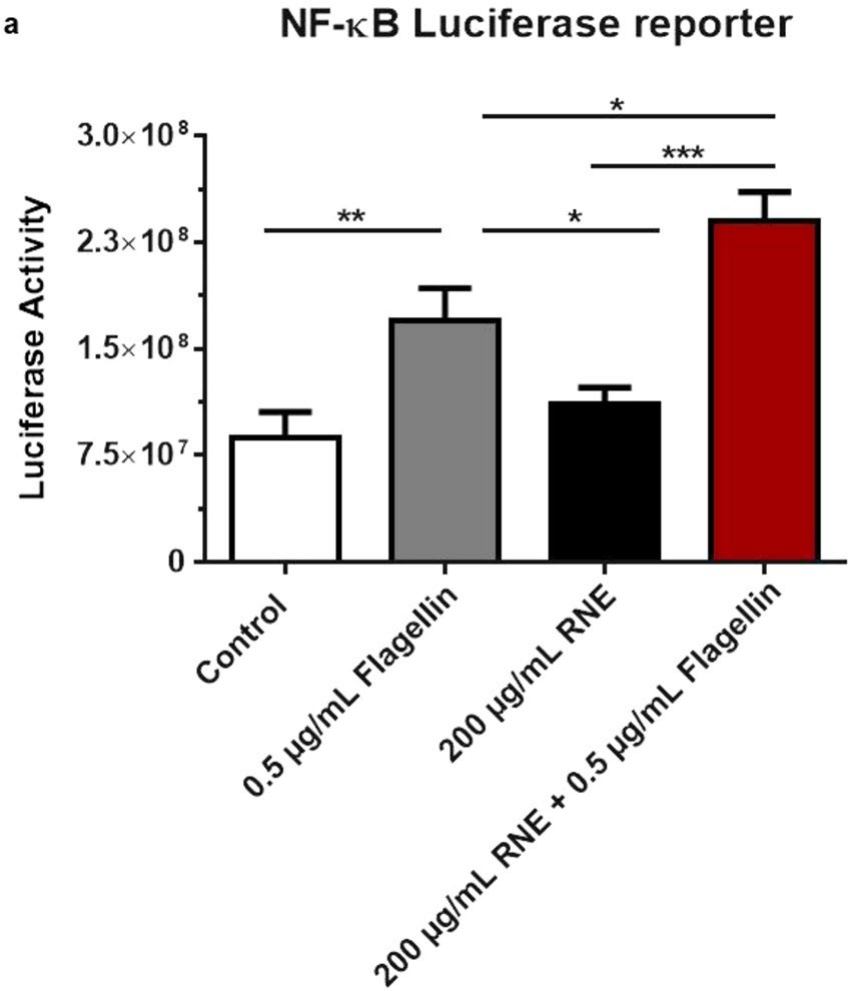
RNE NF-κB pathway in bacterial infection. Luciferase activity of NF-κB in HaCaT transfected with NF-κB luciferase reporter construct was measured. *P* < 0.05, **P < 0.01, ***P < 0.001, Two-way ANOVA analysis analysis. Data are representative of three independent experiments with n = 6 replicates (mean and SEM.).

Finally, the activation of NF-κB was assayed to confirm these observations since TLR5 stimulation leads to NF-κB import inside the nucleus where it acts as transcription factor (Fig. 4a). For that, we took use of NF-κB luciferase reporter assay to monitor the activation of NF-κB. KCs were transfected with pNF-CMV-luc reporter vector prior to stimulation with either Flagellin alone, RNE alone or with both. Enhancement of luciferase activity was observed in Flagellin but not RNE stimulated KCs indicating the activation of NF-κB pathway in case of Flagellin application and confirming the absence of RNE induced NF-κB dependent stress. In accordance with the previous obtained results, the co-stimulation resulted in the highest significant (~two fold) luciferase activity compared to Flagellin simulation alone (Fig. 5).

### RNE is a natural antiproliferative agent

**Figure 6:**
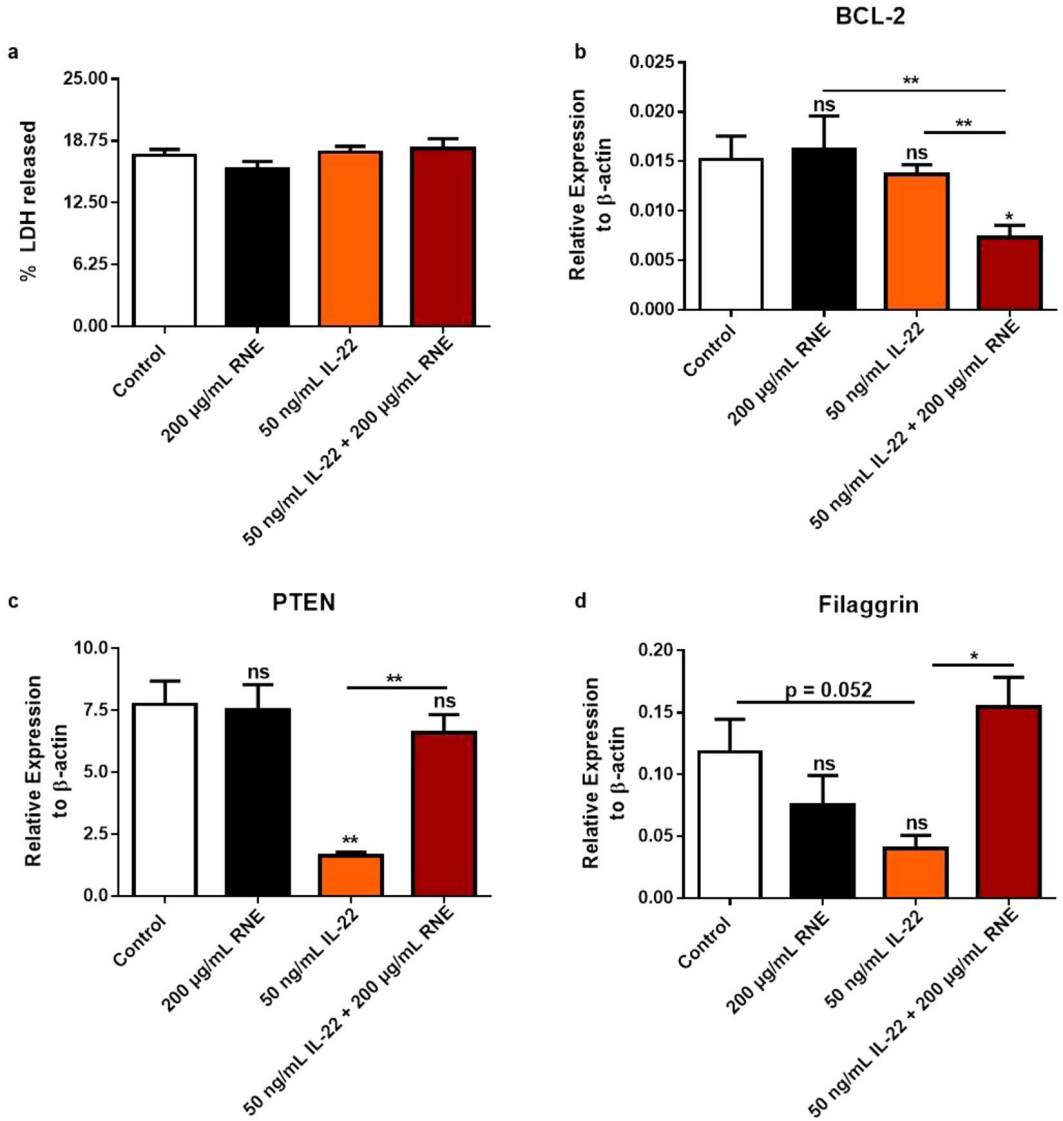
RNE non-toxic natural agents endowed with anti-proliferative activity. HaCaT were treated with or without 50 ng/mL IL-22 and/or 200 μg/mL of RNE for 48 h. The cytotoxicity was evaluated with an LDH assay (**a**) The relative expression of BCL-2 (**b**) PTEN (**c**), and Filaggrin (**d**) were calculated by qRT-PCR. *P < 0.05, **P < 0.01, ***P < 0.001, Two-way ANOVA analysis. Data are representative of two independent experiments with duplicates (mean and SEM.).

To further study the impact of *R*. *nukuhivensis* in skin, we thought to investigate its possible activity in inflamed hyperproliferative skin. IL-22 cytokine, an IL-10 family member up regulated in skin disorders such as psoriasis and atopic dermatitis, was used to stress keratinocytes. Indeed, IL-22 is known to induce KCs hyper-proliferation and to inhibit KCs differentiation^26,44–46^. First, we checked whether the stimulation of KCs with either 50 ng/mL IL-22 alone, RNE alone or co-stimulation with both compounds did induce any cytotoxicity. The LDH assay showed same profile between the different conditions and non-treated cells indicating the absence of cytotoxicity (Fig. 6a). Then, we measured the mRNA levels of PTEN the negative regulator of cellular proliferation, Filaggrin the differentiation marker known to be downregulated in presence of IL-22 and BCL-2 the anti-apoptotic molecule. Accordingly, we showed that IL-22 stimulation resulted in their down-regulation whereas RNE stimulation alone didn’t impact their expression. However, in same experimental conditions, the co-stimulation resulted in PTEN and Filaggrin expression restoration and BCL-2 downregulation (Fig. 6b–d). All these data point the ability of *R*. *nukuhivensis* to limit IL-22 induced uncontrolled proliferation.

## Discussion

The aim of this study is to comprehend the impact of RNE on skin in several contexts. To date, the medicinal cutaneous virtue of this plant remains unknown. In the 50’s Sheuer’s group isolated many alkaloids including serpentinin, ajmaline, sandwicine, sandwicencine, tetraphylline, tetraphyllicin and mauiensine from *Rauvolfia* genus^47^. Alkaloids belonging to the family of Ajmalines common to the genus *Rauvolfia* are exploited in pharmacology due to their anti-arrhythmic, anti-depressive, anti-oxidant and anti-tumoral activities. From *R*. *nukuhivensis*, we isolated derivatives of indolo [2,3-a] quinolizinium (nukuhivensium and N12-methyl-nukuhivensium) and demonstrated their anti-arrhythmic activity, by their action on the hERG potassium channel^8,9^. Despite these biological effects, no study addresses the impact of this plant extract on skin that is highly used by locals. This prompted us to investigate the biological effects induced by RNE stimulation in human keratinocytes.

The first set of data indicates that 24 hours cell stimulation with RNE was not harmful but led to an increased metabolic activity; though the proliferation was not disturbed with any detectable mitochondrial stress. Interestingly, RNE exhibits healing properties since cell treatment with the extract resulted in significant improved wound closure compared to untreated cells. This is likely due to keratinocytes migration, as the RNE treatment did not increase the cell proliferation compare to untreated cells as shown by the negative Ki-67 test. Interestingly, this extract did also improve the wound healing of fibroblasts monolayer (data not shown). All these data highlight that this plant extract has a great potential as ingredient in anti-aging products and treatments by its cellular remodeling effect without disturbing major viability cellular processes.

Marquesan women use RNE as an intimate care, which sounds antimicrobial, or antiseptic-like agent. Previous works showed that this extract lacks of direct antimicrobial activity against the *Candida albicans*, *Aspergillus niger*, *Escherichia coli and Staphylococcus aureus* microorganisms^8^. This prompted us to study nevertheless the impact of RNE on skin barrier function by looking at defensins expression known to be major contributors in innate immunity and immunosurveillance. Our data clearly showed the ability of RNE to upregulate significantly hBD-1, which is expressed constitutively in the terminal layer of the skin and ensures cutaneous immunosurveillance. The inducible hBD-2 and hBD-3 were slightly down-regulated. Hence, RNE reinforces the epithelial barrier by increasing the immunosurveillance defensin rather than the proinflammatory inducible defensins. This result suggested that RNE influences the background regulation of KCs without inducing an immune-associated response.

To go further, we assessed the impact of RNE on the innate immune system. Under normal conditions, RNE up-regulated TLR5 gene expression but did not induce the usual chemokine (CXCL-8) and cytokine (IL-6) production enhanced in response to external pathogens stimuli. Moreover, in presence of an external stress such as the TLR5 agonist Flagellin, RNE strengthened the immune response. This led to a more potent and immediate innate immune response via increased chemokine and cytokine production (e.g CXCL-8 and IL-6) in NF-κB dependent manner. To sum up, RNE seems to act by preparing the immune system of KCs to be more reactive and effective immediately through enhancing its innate immune response by increasing the amount of Toll-like receptors. Therefore, RNE could be qualified as a natural immune adjuvant.

Finally, we investigated the role of RNE in inflamed cells by using an IL-22 stress. RNE was able to oppose IL-22 response, at least in cellular proliferation regulation, by restoration of Filaggrin and PTEN expression. As a matter of fact, PTEN major activity is to negatively downregulate PI3K (phosphatidylinositol-3,4,5-trisphosphate) pathway. PI3K can modulate the activation status of multiple signaling cascades including MAPK, AKT, JNK, ERK, etc.^48–51^. These pathways promote proliferative and uncontrolled cell growth diseases such as cancer and some skin disorders including psoriasis. Hence, our results strongly suggest that RNE modulate the induced Jak-Stat signaling pathway by upregulating PTEN that leads to PI3K, MAPK and ERK pathways inhibition^51^. These data were consolidated by the downregulation of BCL-2 that coincides with PTEN upregulation. For instance, in oral squamous cell carcinomas, tumoregenesis was correlated to the overexpression of BCL-2 and PTEN loss of function. Indeed, the downregulation of the anti-apoptotic BCL-2 limits proliferation^52^. Moreover, since it was demonstrated that the simple derivatives of indolo [2,3-a] quinolizinium have the ability to intercalate in the DNA in a region rich in AT causing proliferation inhibition^53^, we cannot exclude that RNE, containing such indole alkaloids, could also act via modulating the DNA structure. These exciting observations open new tracks to pursue non-toxic natural agents endowed with pro-differentiation properties that can be used as a treatment for skin disorders such as psoriasis as well as uncontrolled IL-22 effects. As a perspective of limiting uncontrolled consumption, we are currently leading experiments to identify the component(s) responsible of these biological activities.

## Materials and Methods

### Plant material and extraction

Bark of *Rauvolfia nukuhivensis* was collected on at Maauu in “Terre Déserte” area on Nuku Hiva Island located at a Marquesas archipelago in French Polynesia at an altitude of 477 meters (identified by Dr Jean-François Butaud). A voucher specimen (JFB 2808) has been deposited at the Herbarium of French Polynesia (PAP, Tahiti Island). After collection at 30 °C, the sample was dried with an air dryer device and stored. The dried bark (50 g) ground into powder, was extracted three times by maceration with 150 mL EtOH during 16 h at room temperature. Then, the solvent was removed to yield the crude extract that was filtered, concentrated and lyophilized to yield *Rauvolfia nukuhivensis* extract called RNE (2.1 g).

### Cell culture and reagents

#### Cell handling

Human keratinocytes HaCaT cells were purchased from AddexBio catalog No. T002000. HaCaT cells were grown Dulbecco’s Modified Eagle’s Meduim (DMEM), with 4.5 g/L Glucose, with L-Glutamine (Lonza, catalog # BE12-604F) supplemented with 10% fetal bovine serum and 1% Penicillin (10,000Units) –streptomycin (10 mg) (Sigma-Aldrich, catalog # P0781) at 37 °C with 5% CO_2_.

#### Cell stimulation

HaCaT cells were seeded at 2.5 × 10^5^ cells per well of 24 well plate or 6 × 10^5^ cells per well of 6 well plate for 24 h to get between 50 to 60% of confluency. One-day prior to stimulation, HaCaT cells were starved by replacing the growth medium with DMEM, with 4.5 g/L Glucose, with L-Glutamine (Lonza, catalog # BE12-604F). Then, the stimulation was achieved in the same medium for 24 h with 200 µg/mL RNE, and/or 0.5 µg/mL Flagellin from *Salmonella enterica* serovar Typhimurium (*S*. *Typhimurium* hereafter) (TLR5 ligand, invivogen catalog # tlrl-stfla) and/or 50 ng/mL recombinant human IL-22 (R&D systems, #782-IL010).

### Cell metabolic activity

Cell Proliferation kit II (XTT- Roche) allows to evaluate the cell metabolic activity by assessing the activity of intracellular oxydoreductases. XTT is a colorless or slightly yellow compound that becomes brightly orange upon reduction by cellular effectors as mitochondrial oxidoreductases. HaCaT cells were seeded in 24 well plates (2.5 × 10^5^ per well) and treated as indicated. XTT assay was performed according to manufacturer’s instructions. Briefly, XTT solution at a final concentration 0.3 mg/mL was added in each well and the incubation was carried out for 4 h at 37 °C. During this incubation, the conversion of the yellow tetrazolium salt XTT by viable cells led to the formation of orange formazan solution. The intensity of coloration was quantified by measuring the absorbance (450 nm) using Victor spectrophotometer (PerkinElmer, Waltham, MA, USA).

### LDH assay

The lactate dehydrogenase (LDH) released upon cell lysis was measured to evaluate cell cytotoxicity. The CytoTox 96^®^ Non-Radioactive Cytotoxicity Assay (catalog # G1780, promega) was used following the manufacturer’s instructions. Briefly, the LDH test quantifies the extracellular lactate dehydrogenase. This enzyme located in the cytosol, is found outside the cell in case of necrosis. The test consists of a colorimetric assay of formazan obtained by the lactate dehydrogenase activity. The ratio of LDH measured in the supernatant over the total LDH (LDH from the supernatant and LDH from the cell lysate), gives the cytotoxic percentage corresponding to the ratio of secreted LDH × 100.

### Flow cytometric analysis for Ki67 staining

HaCaT cells were harvested and spun down to a pellet at 300 g for 5 minutes. The cells were washed with 1 mL of Phosphate Buffered Saline (PBS-1X) (Sigma-Aldrich) and centrifuged at 1500 rpm for 5 minutes. The cells were fixed with 4% *p-*formadehyde at room temperature for 30 minutes. Then, they were pelleted by centrifugation, resuspended with a permeabilization buffer (50 µL of buffer per 1 × 10^6^ cells). Afterwards, cells were centrifuged and stained with 5 µL of Alexa Fluor 700 mouse anti-human Ki-67 antibody (BD Pharmingen, #561277) and isotype control (BD Pharmingen, #557882) followed by room temperature incubation for 30 minutes protected from light. Finally, the cells were washed twice with 2 mL of 0.2% Saponin; resuspended and analyzed with a BD LSR Fortessa Flow Cytometer (BD Biosciences).

### Oxidative stress evaluation

ROS assay was achieved by Fluorometric intracellular ROS kit (catalog # MAK143, Sigma-Aldrich) following the manufacturer’s instructions. To evaluate specifically H_2_O_2_ amount, Fluorometric Hydrogen Peroxide kit (catalog # MAK165, Sigma-Aldrich) was used according to the manufacturer’s instructions.

### *In vitro* keratinocyte wound healing assay

HaCaT cells were grown in a 24 well plate until a monolayer was formed. Subsequently, the grown cells were starved for 24 h before the performance of a scratch with a 200 µL pipette tip in the middle of the well across two opposite edges. Thereafter, the cells were gently washed twice with PBS and stimulated as stated above. Finally, the cell migration was followed in real time by video microscopy using a Zeiss Axiovert 200 M (Carl Zeiss Inc.) fully motorized microscope during 24 hours (scan speed: 1 image every hour). Note that cells were incubated in an atmosphere and temperature controlled chamber at 37 °C and 5% CO_2_. Quantitative analysis of the cell free area was performed using the Axiovision Rel. 4.7 (Carl Zeiss Inc.). The level of wound healing across time (up to 96 h) was evaluated by calculating the percentage of the cell free area at T_x_ divided by the cell free area at the initial state.

### RNA extraction, reverse transcription and real time qPCR

To obtain total cDNA from HaCaT cells, total mRNA was extracted using NucleoSpin® RNA XS kit from Macherey-Nagel following the manufacturer’s instructions. The extracted RNA was reverse transcribed in a two-step protocol using First Strand cDNA Synthesis kit (#K1612) (Thermo Scientific) according to the manufacturer’s instructions. qPCR was carried out with Luna® Universal Probe qPCR Master Mix (Promega, Catalog # M3004S) in LightCycler 480 (Roche). The relative expression of target genes was measured by the comparative CT (critical threshold) method, normalized to housekeeping genes β-actin (Forward: GGGCGCCCCAGGCACCAGGT and Reverse: CGTGCTCGATGGGGTACTTC) and GAPDH (Forward: GAAGGTCGGAGTCAACGGATTTG and Reverse: TGGAGGGATCTCGCTCCTGGA) and determined by the formula 2^−ΔΔCT^. Primers for target genes were purchased from QuantiTect Primer assay Qiagen (check Table 1) unless for Filaggrin (Forward: CAGCTGACAGGCAAGGGC; Reverse: CTGTGAGCTCCTACTGCCTG).

**Table 1:** Quantitect Primer Assay, Qiagen.

Gene Symbol	Assay Name	Cat. no
TLR5	Hs_TLR2_2_SG	QT01009596
PTEN	Hs_PTEN_1_SG	QT00086933
BCL-2	Hs_BCL2_1_SG	QT00025011

### Enzyme Linked Immuno Sorbent Assay

To evaluate the inflammatory response, the culture medium was collected from treated keratinocytes and stored at −80 °C. The protein levels of CXCL-8 and IL-6 were assayed by DuoSet ELISA kit (R&D systems) according to the manufacturer’s instructions.

### Luciferase reporter assays

One-day prior to transfection, HaCaT cells were seeded onto 24 well plates at 1.5 × 10^5^ cells per well. The next day, cells were around 60% of confluence and thus transfection was performed using PTG1 transfection reagent (Polytheragene SAS, Evry, France) with 2.5 μg pNF-CMV-luc reporter vector^54^ at DNA/Polymer weight ratio of 1/6. 4 h after transfection, the medium was replaced with DMEM-High glucose supplemented with 1% FBS overnight. Then, the cells were treated as mentioned previously. Firefly luciferase gene expression was measured using beetle luciferin (Promega # E1601). Luciferase activity was normalized to total protein amount using BCA protein assay kit (Interchim, protein quantitation kit, BCAssays, UP40840A).

### Statistical analysis

All the experiments in the study were reproduced at least two independent different experiments with n = 3 per group. All data are presented as mean ± SEM. Two-tailed student t-test was used to determine significance between two groups. Ordinary two-way ANOVA analysis was used to multiple comparisons for more than two groups. *P*-values less than 0.05 were considered significantly different. (*) P < 0.05, (**) P < 0.01, (***) P < 0.001.

## Supplementary information

Dataset 1

## Data Availability

The datasets generated during the current study are available from the corresponding author on reasonable request.

## References

[1] Stöckigt, J. Chapter 2: Biosynthesis in Rauwolfia serpentina* Modern Aspects of an Old Medicinal Plant. In *The Alkaloids: Chemistry and Pharmacology* **47**, 115–172 (Academic Press, 1995).

[2] Vakil RJ (1955). Rauwolfia serpentina in the treatment of high blood pressure; a review of the literature. Circulation.

[3] Dey A, De JN (2010). Rauvolfia serpentina (L). Benth. ex Kurz.-A Review. Asian J. Plant Sci..

[4] Pathania S, Randhawa V, Bagler G (2013). Prospecting for Novel Plant-Derived Molecules of Rauvolfia serpentina as Inhibitors of Aldose Reductase, a Potent Drug Target for Diabetes and Its Complications. PLoS One.

[5] Girardi C (2015). Herbal medicine in the Marquesas Islands. J. Ethnopharmacol..

[6] Endress ME, Bruyns PV (2000). A revised classification of the Apocynaceae s.l. Bot. Rev..

[7] Ganapaty S, Steve TP, Venkata RK, Neeharika V (2001). A review of phytochemical studies of Rauwolfia species. Indian drugs.

[8] Martin NJ, Prado S, Lecellier G, Thomas OP, Raharivelomanana P (2012). Nukuhivensiums, indolo[2,3-a]quinoliziniums from the marquesan plant rauvolfia *nukuhivensis*. Molecules.

[9] Martin NJ (2015). Indole alkaloids from the Marquesan plant Rauvolfia *nukuhivensis* and their effects on ion channels. Phytochemistry.

[10] Jost X, Ansel J-L, Lecellier G, Raharivelomanana P, Butaud J-F (2016). Ethnobotanical survey of cosmetic plants used in Marquesas Islands (French Polynesia). J. Ethnobiol. Ethnomed..

[11] Abdallah, F., Mijouin, L. & Pichon, C. Skin Immune Landscape: Inside and Outside the Organism. *Mediators Inflamm*. **2017** (2017). 10.1155/2017/5095293 PMC5664322 29180836

[12] Nestle FO, Di Meglio P, Qin J-Z, Nickoloff BJ (2009). Skin immune sentinels in health and disease. Nat. Rev. Immunol..

[13] McKenzie RC SD (1990). Keratinocyte cytokines and growth factors. Functions in skin immunity and homeostasis. Dermal Clin..

[14] Fulton C, Anderson GM, Zasloff M, Bull R, Quinn AG (1997). Expression of natural peptide antibiotics in human skin. Lancet (London, England).

[15] Lehrer R, Ganz T (1999). Antimicrobial peptides in mammalian and insect host defence. Curr. Opin. Immunol..

[16] Harder J, Meyer-Hoffert U, Wehkamp K, Schwichtenberg L, Schröder J-M (2004). Differential Gene Induction of Human β-Defensins (hBD-1, -2, -3, and -4) in Keratinocytes Is Inhibited by Retinoic Acid. J. Invest. Dermatol..

[17] Harder J, Bartels J, Christophers E, Schröder J-M (2001). Isolation and Characterization of Human β-Defensin-3, a Novel Human Inducible Peptide Antibiotic. J. Biol. Chem..

[18] Liu AY (2002). Human β-Defensin-2 Production in Keratinocytes is Regulated by Interleukin-1, Bacteria, and the State of Differentiation. J. Invest. Dermatol..

[19] Niyonsaba F (2007). Antimicrobial Peptides Human b-Defensins Stimulate Epidermal Keratinocyte Migration, Proliferation and Production of Proinflammatory Cytokines and Chemokines. J. Invest. Dermatol..

[20] Lebre MC (2007). Human keratinocytes express functional Toll-like receptor 3, 4, 5, and 9. J Invest Dermatol.

[21] Yao C (2015). Toll-like receptor family members in skin fibroblasts are functional and have a higher expression compared to skin keratinocytes. Int. J. Mol. Med..

[22] Olaru F, Jensen LE (2010). Chemokine expression by human keratinocyte cell lines after activation of Toll-like receptors. Experimental Dermatology.

[23] Gewirtz AT, Navas TA, Lyons S, Godowski PJ, Madara JL (2001). Cutting edge: bacterial flagellin activates basolaterally expressed TLR5 to induce epithelial proinflammatory gene expression. J. Immunol..

[24] Tallant T (2004). Flagellin acting via TLR5 is the major activator of key signaling pathways leading to NF-kappa B and proinflammatory gene program activation in intestinal epithelial cells. BMC Microbiol..

[25] Van Maele L (2010). TLR5 Signaling Stimulates the Innate Production of IL-17 and IL-22 by CD3negCD127+ Immune Cells in Spleen and Mucosa. J. Immunol..

[26] Boniface K (2005). IL-22 Inhibits Epidermal Differentiation and Induces Proinflammatory Gene Expression and Migration of Human Keratinocytes. J. Immunol..

[27] Wolk K (2004). IL-22 increases the innate immunity of tissues. Immunity.

[28] Wolk K (2006). IL-22 regulates the expression of genes responsible for antimicrobial defense, cellular differentiation, and mobility in keratinocytes: A potential role in psoriasis. Eur. J. Immunol..

[29] Sabat R, Ouyang W, Wolk K (2014). Therapeutic opportunities of the IL-22-IL-22R1 system. Nat. Rev. Drug Discov..

[30] Van Belle AB (2011). IL-22 Is Required for Imiquimod-Induced Psoriasiform Skin Inflammation in Mice. J. Immunol..

[31] Fukaya T (2018). Pivotal role of IL-22 binding protein in the epithelial autoregulation of Interleukin-22 signaling in the control of skin inflammation. Front. Immunol..

[32] Ma H-L (2008). IL-22 is required for Th17 cell-mediated pathology in a mouse model of psoriasis-like skin inflammation. J. Clin. Invest..

[33] Zenewicz LA, Flavell RA (2011). Recent advances in IL-22 biology. Int. Immunol..

[34] Xie MH (2000). Interleukin (IL-22), a novel human cytokine that signals through the interferon receptor-related proteins CRF2-4 and IL-22R. J. Biol. Chem..

[35] Kotenko SV (2001). Identification of the functional interleukin-22 (IL-22) receptor complex: the IL-10R2 chain (IL-10Rbeta) is a common chain of both the IL-10 and IL-22 (IL-10-related T cell-derived inducible factor, IL-TIF) receptor complexes. J. Biol. Chem..

[36] Lejeune D (2002). Interleukin-22 (IL-22) activates the JAK/STAT, ERK, JNK, and p38 MAP kinase pathways in a rat hepatoma cell line. Pathways that are shared with and distinct from IL-10. J. Biol. Chem..

[37] Sonnenberg GF, Fouser LA, Artis D (2011). Border patrol: Regulation of immunity, inflammation and tissue homeostasis at barrier surfaces by IL-22. Nature Immunology.

[38] Sonnenberg GF, Fouser LA, Artis D (2010). Functional Biology of the IL-22-IL-22R Pathway in Regulating Immunity and Inflammation at Barrier Surfaces. In. Advances in immunology.

[39] Aujla SJ (2008). IL-22 mediates mucosal host defense against Gram-negative bacterial pneumonia. Nat. Med..

[40] Malhotra N (2016). IL-22 derived from γδ T cells restricts Staphylococcus aureus infection of mechanically injured skin. J. Allergy Clin. Immunol..

[41] Zenewicz LA (2007). Interleukin-22 but not interleukin-17 provides protection to hepatocytes during acute liver inflammation. Immunity.

[42] Zenewicz LA (2008). Innate and adaptive interleukin-22 protects mice from inflammatory bowel disease. Immunity.

[43] Lindemans CA (2015). Interleukin-22 promotes intestinal-stem-cell-mediated epithelial regeneration. Nature.

[44] Dudakov, J. A., Hanash, A. M. & Van Den Brink, M. R. M. Interleukin-22: Immunobiology and Pathology, 10.1146/annurev-immunol-032414-112123 (2015). 10.1146/annurev-immunol-032414-112123 PMC4407497 25706098

[45] Eyerich S (2009). Th22 cells represent a distinct human T cell subset involved in epidermal immunity and remodeling. J. Clin. Invest..

[46] Lowes MA, Suárez-Fariñas M, Krueger JG (2014). Immunology of psoriasis. Annual review of immunology.

[47] Poupon, E. & Nay, B. *Biomimetic organic synthesis*. (Wiley-VCH, 2011).

[48] Meng F (2007). MicroRNA-21 regulates expression of the PTEN tumor suppressor gene in human hepatocellular cancer. Gastroenterology.

[49] Xu L-F (2014). MicroRNA-21 (miR-21) regulates cellular proliferation, invasion, migration, and apoptosis by targeting PTEN, RECK and Bcl-2 in lung squamous carcinoma, Gejiu City, China. PLoS One.

[50] Umemura S (2014). Therapeutic priority of the PI3K/AKT/mTOR pathway in small cell lung cancers as revealed by a comprehensive genomic analysis. J. Thorac. Oncol..

[51] Carracedo A, Pandolfi PP (2008). The PTEN-PI3K pathway: Of feedbacks and cross-talks. Oncogene.

[52] Rahmani A, Alzohairy M, Babiker AY, Rizvi MA, Elkarimahmad HG (2012). Clinicopathological significance of PTEN and bcl2 expressions in oral squamous cell carcinoma. Int. J. Clin. Exp. Pathol..

[53] Abarca B (2014). Efficient synthesis of an indoloquinolizinium alkaloid selective DNA-binder by ring-closing metathesis. Org. Lett..

[54] Gonçalves C (2009). An optimized extended DNA kappa B site that enhances plasmid DNA nuclear import and gene expression. J. Gene Med..

