# Supplementary material for: R. nukuhivensis acts by reinforcing skin barrier function, boosting skin immunity and by inhibiting IL-22 induced keratinocyte hyperproliferation
Source: Sci Rep. 2019 Mar 11;9:4132. doi: 10.1038/s41598-019-39831-w (PMC6411885; doi:10.1038/s41598-019-39831-w)
Supplement: Supplementary file 1 — Dataset 1 [file 41598_2019_39831_MOESM1_ESM.pdf]

Title: *R. nukuhivensis* acts by reinforcing skin barrier function, boosting skin immunity and by inhibiting IL-22 induced keratinocyte hyperproliferation.

Florence Abdallah[1], Gaël Lecellier[2], Phila Raharivelomanana[3], Chantal Pichon[1,4*].

[1]Centre de Biophysique Moléculaire, CNRS-UPR4301, 45071 Orléans, France.
[2] Université de Paris-Saclay UVSQ, 55 Avenue de Paris, 78000 Versailles, France
[3] Université de la Polynésie Française, UMR 241 EIO, 6570 - 98702 Faa'a - Tahiti, Polynésie Française.
[4] Université d'Orléans, Collegium Sciences et Techniques, 45100 Orléans, France.

*Corresponding author: chantal.pichon@cnrs.fr

Materials and Methods

Cell culture and stimulation. As described in materials and methods.

Seahorse test. The assessment of mitochondrial function was achieved with Seahorse XF Cell Mito Stress Test Kit (Agilent, # 103015-100) following the manufacturer's instructions. Briefly, this test can assess multiple parameters implicated in mitochondrial respiration including basal respiration, ATP production-coupled respiration, maximal and reserve capacities and non-mitochondrial respiration via inhibitors addition of each parameter. One day prior to test performance, the cells were seeded into the wells of an XF cell culture microplate. Then, the cell culture medium was replaced with bicarbonate free medium with or without 200 µg/mL plant extract. After the cartridge calibration, the plate is inserted into the instruments were measurements are achieved.

Figure Legends

Figure S1: Dose-response curve to RNE stimulation. The evolution of the cellular viability upon RNE stimulation over times (up to 48h) was followed by an XTT assay. *P<0.05, **P<0.01, ***P<0.001, Two-way ANOVA analysis. Data are representative of two independent experiments with duplicates (mean and SEM.).

Figure S2: Mitochondrial and non-mitochondrial bioenergetics upon RNE application. Representative scheme of the seahorse assay (a).The oxygen consumption rate (OCR) in mitochondrial and non-mitochondrial respiration was measured as pmol/min (b and c) and extracellular acidification rate (ECAR) for non-mitochondrial respiration was measured as pH/min (d) in presence or absence of 200 μg/mL of RNE.

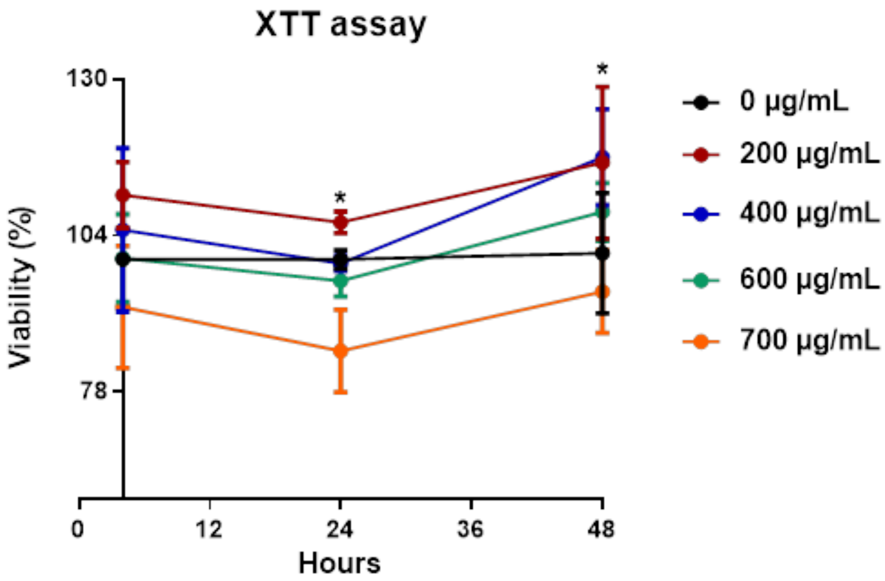
XTT assay
130
104
78
Viability (%)
0
12
24
36
48
Hours
*
*
0 µg/mL
200 µg/mL
400 µg/mL
600 µg/mL
700 µg/mL

Figure S1.

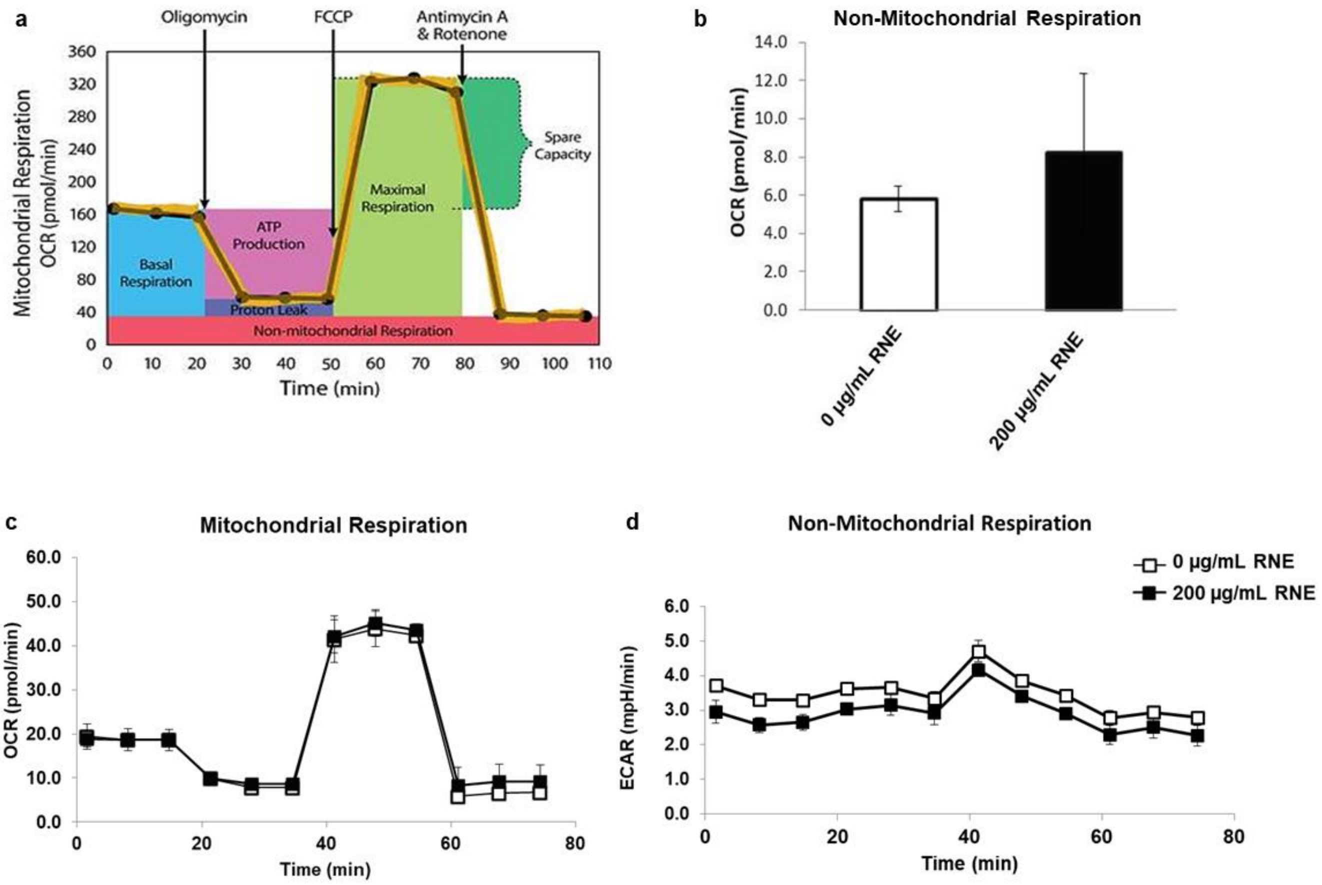
a
Oligomycin
FCCP
Antimycin A
& Rotenone
Mitochondrial Respiration
OCR (pmol/min)
Spare
Capacity
Maximal
Respiration
ATP
Production
Basal
Respiration
Proton Leak
Non-mitochondrial Respiration
Time (min)
b
Non-Mitochondrial Respiration
OCR (pmol/min)
0 µg/mL RNE
200 µg/mL RNE
c
Mitochondrial Respiration
OCR (pmol/min)
Time (min)
d
Non-Mitochondrial Respiration
0 µg/mL RNE
200 µg/mL RNE
ECAR (mpH/min)
Time (min)

Figure S2.
